# Variation of the Myelin Oligodendrocyte Glycoprotein gene is not primarily associated with multiple sclerosis in the Sardinian population

**DOI:** 10.1186/1471-2156-8-25

**Published:** 2007-05-17

**Authors:** Maria Giovanna Marrosu, Raffaele Murru, Gianna Costa, Maria Cristina Melis, Marcella Rolesu, Lucia Schirru, Elisabetta Solla, Stefania Cuccu, Maria Antonietta Secci, Michael B Whalen, Eleonora Cocco, Maura Pugliatti, Stefano Sotgiu, Giulio Rosati, Francesco Cucca

**Affiliations:** 1 Centro Sclerosi Multipla, Dipartimento di Scienze Neurologiche e Cardiovascolari, University of Cagliari, Italy; 2 Istituto di Neurologia Clinica, Facoltà di Medicina e Chirurgia, University of Sassari, Italy; 3 Dipartimento di Scienze Biomediche, University of Sassari, Italy

## Abstract

**Background:**

Multiple sclerosis (MS) is consistently associated with particular HLA-*DRB1*-*DQB1 *haplotypes. However, existing evidence suggests that variation at these loci does not entirely explain association of the HLA region with the disease. The *MOG *locus is a prime positional and functional candidate for such additional predisposing effects but the analysis is complicated by the strong, albeit labyrinthine pattern of linkage disequilibrium in the region. Here we have assessed the association of *MOG *variation with MS in the Sardinian population to see if it represents an independent contributor to MS predisposition.

**Results:**

After re-sequencing the *MOG *gene in 21 healthy parents of MS patients we detected 134 variants, 33 of which were novel. A set of 40 informative SNPs was then selected and assessed for disease association together with 1 intragenic microsatellite in an initial data set of 239 MS families. This microsatellite and 11 SNPs were found to be positively associated with MS, using the transmission disequilibrium test, and were followed up in an additional 158 families (total families analysed = 397). While in these 397 families, 8 markers showed significant association with MS, through conditional tests we determined that these *MOG *variants were not associated with MS independently of the main *DRB1*-*DQB1 *disease associations.

**Conclusion:**

These results indicate that variation within the *MOG *gene is not an important independent determinant of MS-inherited risk in the Sardinian population.

## Background

Multiple sclerosis (MS) is a serious chronic inflammatory and demyelinizating disorder of the central nervous system which results from an autoimmune attack on components of the oligodendrocyte cell [1]. The disease is more common in European and European-derived populations. Within Europe it shows a north-south gradient, with the notable exception of the Mediterranean island of Sardinia, which has one of the highest prevalences worldwide [2]. The disease risk for a monozygotic twin of an affected patient is about 30%, showing a quick fall-off rate with decreased genetic relatedness to affected individuals. These data, and the increasing incidence of disease reported in some populations over the last few decades [3,4], suggest that the chance of this inflammatory process occurring depends on the complex interplay between a polygenic trait and unknown environmental factors influencing the penetrance of susceptibility genes [5,6]. MS has been found to be consistently associated with specific HLA class II variants and notably with the HLA-DRB1*1501-DQB1*0602 haplotype, which represents the main risk factor for disease occurrence in different ethnic backgrounds [7-11]. The relative contribution of variation at the *DRB1 *and *DQB1 *loci to disease predisposition is still not completely clear although some studies based on cross-comparing rare *HLA DRB1-DQB1 *haplotype splits have indicated that the main contribution comes from variation at the *DRB1 *locus [11]. Analysis of large data sets from Finland and Canada have also suggested that in those populations, any important additional modifiers of MS susceptibility were likely to be contained in the regions close to *DRB1 *[12]. In Sardinia, the HLA-DRB1*1501-DQB1*0602 haplotype is rare but is still significantly positively associated with MS together with an additional 4 haplotypes including DRB1*1303-DQB1*0301, DRB1*0405-DQB1*0301, DRB1*0301-DQB1*0201 and DRB1*0405-DQB1*0302 [13]. Some of these haplotypes, such as DRB1*0301-DQB1*0201 are also associated with MS in some non-Sardinian populations [11,14,15] but not in others [8,9,16-20]. The presence of HLA non-*DRB1-DQB1 *predisposing effects related to a different distribution of extended HLA-DRB1*0301-DQB1*0201 haplotypes in different populations can explain these findings. Indeed, some studies have suggested that within the HLA region there are further independent predisposing effects determined by as yet unidentified non-*DRB1*-*DQB1 *variants [13,21-24]. However, strong linkage disequilibrium (LD) between the variants contained in the HLA region makes it difficult to detect which polymorphisms, outside the exon 2 sequences of the DR/DQ loci but within the HLA region, further influence disease risk. Within the HLA region, the *MOG *gene is a prime candidate for additional MS associations. This gene is located, 2.9 Mb telomeric of the *DRB1 *locus, in a chromosome interval which has shown some evidence of association with MS independent of *DRB1-DQB1 *[13]. Furthermore, the rodent ortholog of this gene encodes for an autoantigen which triggers autoimmune responses in experimental models of disease [25,26]. Moreover, in MS patients both T-cell and antibody responses against this protein have been detected [27,28] and an aetiologic role of anti-*MOG *antibodies has been suggested in acute lesions of MS patients [29], although this role is controversial [30,31]. These findings could be consistent with a model in which specific polymorphisms in the *MOG *gene could determine amino acid variation or differences in the level of expression of this protein in the central nervous system and affect immune responses against it, thus acting as primary aetiologic determinants of disease pathogenesis.

A few studies have tested the association between *MOG *polymorphisms and disease, with conflicting results. Some of these studies indicated a potential role of *MOG *[32,33] while others failed to find any evidence of primary association with MS [34,35,12]. However, none of these studies analysed the *MOG *gene comprehensively, nor assessed the association of genetic variation of this gene in the context of extended *DRB1-DQB1-MOG *haplotypes.

The aim of this study was to establish the SNP content of the *MOG *gene in the Sardinian population and use this information together with appropriate conditional association tests to assess the individual contribution of the *MOG *gene to MS inherited risk to determine if it is an important independent risk locus.

## Results and discussion

**Figure 1:**
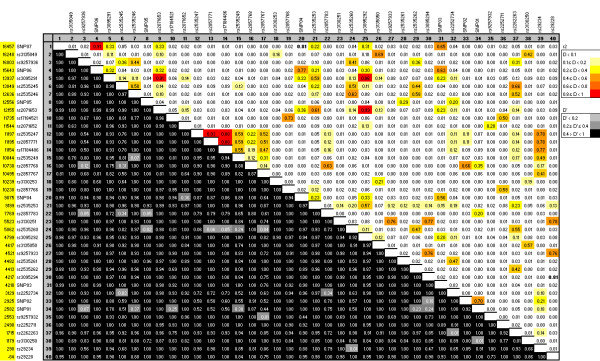
Distribution of *r2 *and *D*' intermarker values (above and below the diagonal respectively) for 40 SNPs analysed in 478 parents of Sardinian MS patients.

In order to assess the association of *MOG *variation with MS we first established polymorphism content of the *MOG *gene in the Sardinian population by resequencing the gene in 21 healthy parents of MS patients. The primers used are listed in Table 1, and technical details are reported in the Methods section. We detected 134 SNPs, 101 of which had already been reported [36] and 33 were novel. A set of 40 informative SNPs was then selected to be tested for disease association (see Methods). These were initially genotyped in a data set of 239 MS families. To identify the haplotype-tagging patterns, we computed the intermarker *D*' and *r2 *values of these 40 informative SNPs, computed for 478 parents of the MS patients, shown in Figure 1. The physical positions, with base 1165 corresponding to the translation start site of *MOG*, and other relevant information are indicated in Table 2. *D*' values indicate that these SNPs are part of the same linkage disequilibrium block (average 0.97 between contiguous marker pairs) that, as we have previously shown, encompasses HLA class I and III sub-regions and also includes the HLA class II *DR *and DQ loci [37,13]. As expected, considering selected SNPs were chosen in order to avoid genetically redundant markers, the *r2 *values showed relatively low values with an average of 0.13 between contiguous marker pairs (Figure 1).

**Table 1:** 

Primers	Forward 5'-3'	Reverse 5'-3'	TM
1	atgcaacagggagaaagagc	gaccagcattggtagcaggt	57°C
2	tggacagcacggctctaaat	tggacagcacggctctaaat	58°C
3	ggtctggtgatgacagttgtg	ggaagggaggcatgtcagta	60°C
4	tgtattttagtacagacggggtttc	tggtgcctgattatgcagag	60°C
5	gcttagcagtgcctgtccat	gccaaattgctgcctattct	58°C
6	agggtggaagatctcagaag	caaagcgctgggattatg	57°C
7	gctgaggcaggagaatcact	ttggccacccaacagttaat	59°C
8	aaataaaaaggaagaagaagaaga	ccacccaacagttaataccta	57°C
9	ggcagcaatggaattgaaag	cccacagtgctgggattac	58°C
10	ccaggggtttgcagttacag	gcttattcactaggaccaagcac	57°C
11	ttgcagtgagccaaaatcc	tcacccatcatgcaaaacat	63°C
12	tgacctcaggtgatccactc	ggtgaaactgagggagtttg	62°C
13	tgagaccctctccagtttgc	agacgaagaggtgggaacag	60°C
14	tcacatcttacaaactcaacaaaaa	ccaggagggttgcttaggta	59°C
15	ccagtctggcctcgagaact	tcacaaatattggccaggtg	59°C
16	ccagtcctgtaggtgctaaa	cttagccctgaaggaaaaat	56°C
17	tctaaatcactagcatttcctg	gaaagcattccgtaagatgt	58°C
18	tgggttcagaagttcttctcacta	ccccataccctccttgctac	62°C
19	gccagagcaggaagagtc	cgttgccactttgtttatc	56°C
20	gctactcactaaatgttcagctcct	cttgttcccccaggtgact	62°C
21	ctccaccggacttttggtaa	ggtgctcttctgttgccatt	57°C
22	gaattgacccctcaaggaca	aggcagaggtttcagtgagc	61°C
23	ggcagagttttgctcttgtca	tggtgaaaccccgtctctac	58°C
24	agtgcaggggcaggatct	gcatggggacagaggaataa	58°C
25	ccttttctcttcattttcccatt	ggatgagaggaaggaaagattc	59°C
26	tgcctgctttagaatgttttcc	agacaggtgcgtggataagg	59°C
27	aagttttcataagcacacttctaacc	tctctccagacaccagaagg	61°C

**Figure 2:**
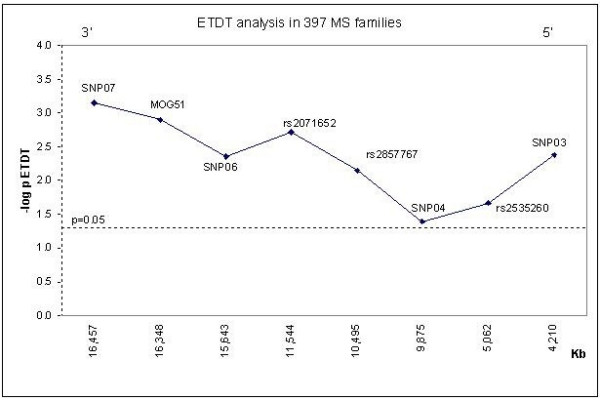
ETDT analysis in 397 MS families. Single-point association of 1 microsatellite and 8 SNP markers with MS within the *MOG *gene. Distances are given in Kb proceeding from the most centromeric marker, SNP07, to the most telomeric, SNP03. Association is presented as the negative log of the *P*-value for the extended transmission disequilibrium test (ETDT). The bold horizontal line corresponds to the threshold significance of 0.05

We then assessed disease association of the 41 variants of interest, including these 40 SNPs of interest and the *MOG51 *microsatellite, in a sample set of 239 families using the extended transmission disequilibrium test (ETDT). Without taking linkage disequilibrium with variation at the HLA-*DRB1*-*DQB1 *loci into account, 11 SNPs -SNP07 (novel), SNP06 (novel), SNP05 (novel), rs2071652, rs2857767, SNP04 (novel), rs2535260, rs2535261, SNP03 (novel), rs2252734, rs29228 and the *MOG51 *microsatellite showed evidence of association at a significance level of 5% with MS (data not shown). We then evaluated MS association of these markers in an additional 158 MS families (total n = 397 families). In this larger sample set, 8 markers showed some evidence of association at 5% significance level (Figure 2) with one haplotype defined by variants at rs2071652 (allele C), rs2857767 (allele C) and rs2535260 (allele C) showing the strongest disease association (*P *= 3.29 × 10^-6^). This level of significance was slightly lower than that shown by association of HLA-*DRB1 *(*P *= 7.9 × 10^-7^) and -*DQB1 *in the same sample set (*P *= 1.3 × 10^-7^). The transmission data of these variants individually and in the context of the three locus *DRB1-DQB1-MOG *haplotypes are shown in the online appendix along with the *D*' values among *MOG *variants and the various *DRB1-DQB1 *haplotypes. The eight disease associated variants are essentially part of two main LD blocks formed, on the one hand by rs2071652, rs2857767 and rs2535260 and on the other hand by the other five variants. From these data it is evident that while SNP07, SNP06; SNP04, SNP03 are in positive LD only with the DRB1*0301-DQB1*0201 haplotype, SNPs rs2071652, rs2857767 and rs2535260 occur frequently (albeit at different degrees of positive LD from strong to modest) and show a significant association with more than one *HLA-DRB1-DQB1 *haplotype. The co-occurrence of these *MOG *variants with different *DRB1-DQB1 *haplotypes could just be a chance event or could be due to any of the various forces which impact LD [37]. Importantly, we also assessed the disease association of *MOG *alleles which individually showed significant evidence of association with MS together with the other *DRB1-DQB1 *haplotypes grouped (that is, all *DRB1-DQB1 *haplotypes which did not show any evidence of significant association with MS). These extended *DRB1-DQB1*-*MOG *haplotypes showed a consistent negative transmission from parents to affected patients (see Additional file 1). This suggested that *MOG *disease associations were most likely driven by *DRB1-DQB1 *variation.

Next, we wanted to directly assess whether these *MOG *associations were independent from *DRB1-DQB1 *associations. To this end, we analysed the overall effect of allelic and haplotype variation at the *MOG *gene using the conditional ETDT (CETDT) [38]. Using this conditional analysis, which takes into account linkage disequilibrium between overall *MOG *variation and overall variation at the *HLA-DQB1 *and -*DRB1 *loci, *MOG *associations were no longer significant. Conversely, the overall associations of *DRB1-DQB1 *haplotypes, taking into account the most associated *MOG *haplotype, gave a *P *value of 9.7 × 10^-3^.

**Figure 3:**
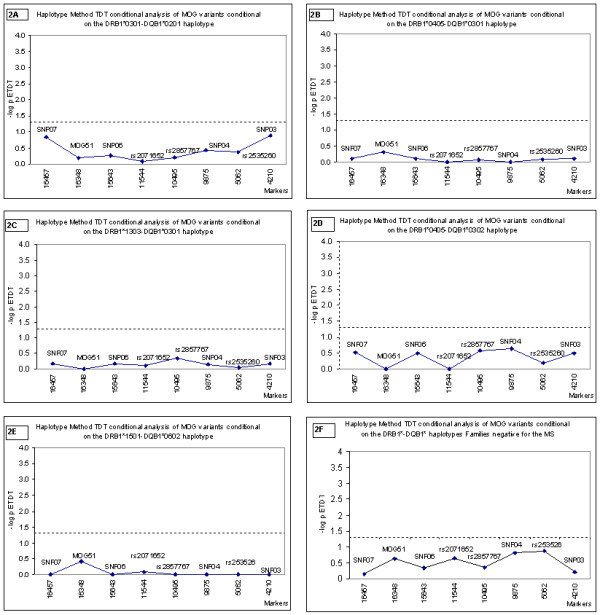
Association analysis of 8 *MOG *variants with MS conditional on *DRB1-DQB1 *haplotypes using the Haplotype Method TDT Association analysis of 8 *MOG *variants with MS conditional on *DRB1-DQB1 *haplotypes using the Haplotype Method-Transmission Disequilibrium Test HM-TDT [39]. The 8 *MOG *variants are the same shown in Figure 1 while the 5 predisposing *DRB1-DQB1 *haplotypes refer to DRB1*0301-DQB1*0201 (2A), DRB1*0405-DQB1*0301 (2B), DRB1*1303-DQB1*0301(2C), DRB1*0405-DQB1*0302 (2D) and DRB1*1501-DQB1*0602 (2E), with DRB1*-DQB1* families negative for MS haplotypes that showed prior evidence of association with MS (2F) [13]. Association is presented as the negative log of the *P*-value for the extended transmission disequilibrium test (ETDT). The bold horizontal line corresponds to the threshold significance of 0.05

We also evaluated the relative transmission of the *MOG *alleles and haplotypes according to which specific *DRB1-DQA1-DQB1 *haplotypes they were on (Figure 3). In order to carry out these analyses we used the haplotype method (HM)-TDT (Methods) [39]. Using this test we initially evaluated association of specific variants of *MOG*, taking into account linkage disequilibrium with the *HLA-DRB1-DQB1 *haplotypes for which there is prior evidence of association with MS in the Sardinian population (i.e. DRB1*1303-DQB1*0301, DRB1*0405-DQB1*0301, DRB1*0301-DQB1*0201, DRB1*1501-DQB1*0602 and DRB1*0405-DQB1*0302 haplotypes) [13]. Again, allelic variation at the *MOG *locus did not significantly affect the transmission of these predisposing *DRB1-DQB1 *haplotypes (Figure 3). Also the *MOG *haplotype defined by rs2071652, rs2857767 and rs2535260 did not produce significant evidence of primary association when analysed with the HM-TDT conditional on *DRB1-DQB1 *positively associated haplotypes (data not shown). Moreover, we also conducted the HM-TDT to assess whether these *MOG *variants were influencing association of the non-predisposing *DRB1-DQB1 *haplotypes grouped. No significant heterogeneity in the association of *DRB1-DQB1 *haplotype conditional on *MOG *was seen, again suggesting that the initial observations of association with MS risk were likely to be only a consequence of LD with the DR-DQ mediated MS risk. This is shown in Figure 3, Graph F.

Considering our results globally, they provide evidence that haplotypes identical at the *DRB1*-*DQB1 *loci but different at the *MOG *locus do not have different associations with MS. Thus the significant association of *MOG *alleles and haplotypes appear to be secondary to hitchhiking effects due to variation at the *DRB1*-*DQB1 *loci. In interpreting these results it should be considered that the HLA region as a whole confers only a relatively modest contribution to the familial clustering and to the individual inherited risk of MS. Thus, the statistical power required to rule out a rare disease variant not detected in our resequencing, or even more dramatically, one that only slightly modulates the disease risk conferred by *HLA-DRB1-DQB1 *variants would require unrealistic sample sizes which exceed those used in this study. Still, our results and conclusions are consistent with those obtained in other populations [40], although there is still the suggestion that other non-DR-DQ genes may convey an independent risk to MS [41,42,12,24].

The genetic results presented here have relevant mechanistic biological implications because they suggest that the protein product of the candidate gene *MOG *is unlikely to represent the primary autoantigen in human MS and thus cannot explain the target organ specificity observed in this disease. Contrast these data with the primary and consistent association detected with variation in the insulin gene (*INS*) region in type 1 diabetes and the epistatic deviations from a multiplicative model detected in the joint analysis of HLA class II and *INS *[43].

## Conclusion

From our results we can conclude that variation at the *MOG *gene does not provide a significant independent contribution to the inherited risk for MS in the Sardinian population. The data suggests that the protein product of *MOG *is unlikely to represent the primary autoantigen in human MS.

## Methods

### Study participants

In the study we considered a sample set of 397 independent MS families, which included 391 trios families (one affected child and both healthy parents), 4 multiplex families (more than one affected sibling and their healthy parents) and 2 vertical families (one parent and one affected offspring), all being at least third generation Sardinian. This data set of 397 MS families is a proper subset of the 490 Sardinian MS families described previously [13]. All patients met the criteria of definite MS [33]. The average age of onset for MS was 28.9 years (range 6 – 51 years), and the female/male ratio was 2.3. The disease course was relapsing-remitting in 65%, secondary progressive in 27% and primary progressive in 8% of patients. All families had previously been analysed at the *DRB1-DQA1-DQB1 *loci [13].

### Power calculations

To examine the feasibility of verifying whether variants in the *MOG *gene, or in strong LD with it could explain the association peak that was previously observed in Sardinia [13], we focused on the parent-affected child trios that we had already studied during the initial examination of the role of the HLA in MS susceptibility. We postulated that our search would be for a fairly common variant (in line with the frequency of the predisposing *DRB1-DQB1 *haplotypes) with a maximal relative risk of 1.9 and using a disease prevalence of 0.0014 (corresponding to the prevalence in Sardinia). The strength of LD between the *DRB1-DQB1 *locus and *MOG *was estimated at a mean value of *r*^2 ^= 0.3 as previously shown [13]. Purcell and Sham power calculations for our 391 trios gave for an alpha value of 0.10 a power of 0.83 [44]. It should be noted that for the conditional analyses, in the case of conditioning on the absence of high-risk HLA haplotypes, the trio size is 121 and the corresponding power is reduced to 0.42 for the same alpha criteria.

### MOG polymorphism analysis

We resequenced the *MOG *gene in 21 parents of MS patients heterozygous for the 226 bp allele at the *MOG51 *microsatellite locus, which was found to be significantly associated with MS [13]. This selection of informative parents increases the chance of detecting relevant polymorphisms. 37 overlapping fragments covering the whole gene (8 exons and 7 introns) and -384 and 545 bp 5' and 3' from the transcriptional unit were amplified and capillary sequenced (Megabace 1000). Overall, 16,561 bp were sequenced. Primers were designed according to the published human genomic DNA sequence [34] and are listed in Table 1). PCR was carried out in a final volume of 25 μl using a specific combination of primers and 5 μl of 4 ng/μl genomic DNA. After an initial denaturation of 4 minutes at 95°C, there were 37 cycles at 95°C for 30 seconds, at the specific TM primer for 30 s, 72°C for 30 s and a final extension at 72°C for 7 minutes.

A set of 40 informative SNPs was selected and dot-blot genotyped in the family sample set, using the set of primers listed in Table 1 and the SSO probes reported in Table 2. Selection of these SNPs was based on the patterns of LD (in order to provide good map coverage and avoid genotyping and testing genetically redundant variants), on the position in the gene (in order to maximise the chance of testing functionally relevant variants) and on the minor allele frequency (MAF) (in order to exclude particularly rare variants for which we did not have sufficient statistical power in the subsequent association stage).

**Table 2:** 

Position	MAF (%)	SNPs N.			PCR (bp)		T.A.	Primer set (from Table 1)	SSO probe
-84	9.9	1	rs29228	5'	613	A/G	57°C	1	cggacaaaaAcagaaatgt
290	5.6	2	rs29234	5'	525	A/C	58°C	2	agctgagccAagaggtgag
1179	6.0	3	rs3130250	Ex1	762	G/A	60°C	3	agcttatcGagaccctct
1715	23.0	4	rs2262263	Intr1	509	A/G	60°C	4	cccgcctcAgcttcccaa
2498	7.3	5	rs2252711	Intr1	508	T/C	58°C	5	gagagacagTtaaagtaga
2553	4.2	6	rs9257932	Intr1	408	T/C	57°C	6	ggcagcaatTtggccaagt
2582	1.6	7	SNP01(novel)	Intr1	408	G/A	57°C	6	aggcccataGgaggattca
2925	1.7	8	SNP02(novel)	Intr1	706	A/G	59°C	7	attagctggAtgcggcggt
3129	22.8	9	rs2252734	Intr1	568	C/G	57°C	8	gaagaaCaattgcaatc
4210	12.6	10	SNP03(novel)	Intr2	642	G/A	58°C	9	gcccagcgccgtGgctc
4217	10.5	11	rs3095294	Intr2	642	A/G	58°C	9	ctcacAcctgtaatccca
4413	14.5	12	rs2535262	Intr2	507	G/A	57°C	10	gtgaacccGgaagcggag
4482	17.7	13	rs2535261	Intr2	507	G/A	57°C	10	cagcgagactccGtctca
4521	9.8	14	rs9257933	Intr2	507	G/A	57°C	10	gtatttgtgagcgcGcac
4617	7.0	15	rs3135050	Intr2	580	T/C	63°C	11	gagatgtcacTttttggc
4799	35.9	16	rs3095292	Intr2	580	G/A	63°C	11	ccgagtagctgGgattac
5062	20.9	17	rs2535260	Intr2	584	C/G	62°C	12	aggtgaagcCgatggagg
5523	10.6	18	rs3130251	Intr2	518	C/T	60°C	13	cacctataaCcccaaaac
7769	1.1	19	rs2857783	Intr2	569	T/C	59°C	14	acttgaaagTaaaggtag
9199	47.4	20	rs2535253	Intr2	591	A/C	59°C	15	attacaggaAtgtgccacc
9875	16.3	21	SNP04(novel)	Intr2	404	A/G	56°C	16	ccttgagggActtcagat
10230	10.3	22	rs2857766	Ex3	493	G/C	58°C	17	tcctgcagatcactGttg
10239	1.1	23	rs3130253	Ex3	493	G/A	58°C	17	ttggcctcGtcttcctct
10495	4.4	24	rs2857767	Intr3	573	C/G	62°C	18	taactatgCcatatagtaa
10738	1.3	25	rs2857768	Intr3	573	T/C	62°C	18	ttgtttcctcTttctccat
10844	6.7	26	rs2535249	Intr3	573	A/G	62°C	18	tctccccaAtgccagagca
11154	4.5	27	rs17184486	Intr3	493	A/G	56°C	19	gattctccAagcttcagtt
11195	5.3	28	rs2857771	Intr3	493	C/T	56°C	19	aacatcctCcctcctaaat
11197	5.3	29	rs2535247	Intr3	493	C/G	56°C	19	aacatcctccCtcctaaat
11544	12.9	30	rs2071652	Intr3	740	C/T	62°C	20	ccaggctgCagagaaatag
11735	11.6	31	rs17184521	Intr4	740	G/A	62°C	20	aaatggtcccGttcttgga
12155	36.3	32	rs2071653	Intr5	583	C/T	57°C	21	tacaaaaaCgttatcttat
12556	9.2	33	SNP05(novel)	Intr5	614	C/A	61°C	22	cagatggtaCcttctctga
12636	17.6	34	rs2535246	Intr5	614	T/G	61°C	22	cacagagTtctatcgtacg
13049	22.2	35	rs2535245	Intr5	545	T/C	58°C	23	ctcagcctccTgagtagct
13937	35.8	36	rs3095291	Intr5	713	G/C	58°C	24	cacgcccaGctaattttta
15643	13.9	37	SNP06(novel)	Ex8	527	G/A	59°C	25	tgtgaaggGaaggaagagg
16003	11.8	38	rs9257936	Ex8	593	C/T	59°C	26	gataCgagttttggccggg
16248	6.2	39	rs3135049	Ex8	593	C/T	59°C	26	agctgagatcgCgccactg
16457	13.8	40	SNP07(novel)	3'	569	A/G	61°C	27	attaccccagAggtcagtc

In addition to these SNPs, in this manuscript we also assessed the association of MS with 1 intragenic microsatellite, *MOG51 *[34], whose primer sequences have been previously reported [13].

### Statistical analysis

Single-point disease association of individual *MOG *variants as well as *HLA-DRB1*, *DQB1 *alleles was evaluated using ETDT [45]. This test takes into account the transmission or non-transmission of alleles of a locus relative to those present on the other parental chromosome. In the case of multiallelic markers, the ETDT takes multiple alleles into account and obtains a global *P *value indicative of the degree of significance of the association with the disease at each individual locus. In order to distinguish primary associations from those due to linkage disequilibrium at the various assessed loci, we used a variant of the ETDT, called CETDT [38]. This test allows us to analyse the overall effect of one locus, in our case *MOG*, taking the overall association of variation at other loci into account; in this study *DRB1 *and *DQB1*. In order to study the transmission of specific *MOG *alleles, conditioned on alleles or haplotypes at the *DRB1-DQB1 *disease loci, we used a TDT variant of the haplotype method [46]; the HM-TDT [39]. This method tests the null hypothesis of equality of transmission of haplotypes identical at one variant or combination of variants, but different at another closely-linked test locus [39]. If there is heterogeneity in the transmission of two marker haplotypes identical at a predisposing marker (variant A) but different at a putative predisposing marker at another site (variant B), then this is evidence that variant A does not entirely explain disease predisposition and that variant B itself, or another marker in linkage disequilibrium with variant B, is influencing the transmission of variant A and, thus, disease susceptibility [39].

LD between individual SNPs at the *MOG *gene was assessed computing the *D*' and *r2 *values. Both measures are built on the basic pairwise-disequilibrium coefficient, *D*, described by Lewontin [47], representing the difference between the probability of observing two marker alleles on the same haplotype and observing them independently in the population. The *D *and normalised disequilibrium *D*', or |*D*/*Dmax*|, values were computed on parental haplotypes using the software package [48]. A value of *D*' equal to 0.0 implies complete independence, whereas 1.0 implies that all copies of the rare allele occur exclusively with one of the two possible alleles at the other assessed marker. *R2*, computed using a programme written by Jason Cooper, assesses not only LD between two markers but also the degree of matching between all allelic frequencies of both SNPs [49], thus encompassing the effects of both *D *and all marker allele frequencies. As for *D*', an *r2 *value of 0.0 also implies independence, but *r2 *= 1.0 occurs only when the marker loci have identical allele frequencies and every occurrence of an allele at each of the markers perfectly predicts the allele at the other locus (i.e. in the presence of genetically indistinguishable makers). The two LD measures have different features: *D*' levels are useful to define the extent of LD, for instance to establish the presence of the so-called LD blocks along chromosome intervals. Instead, *r2 *are useful to establish marker informativity and the degree of genetic redundancy between selected SNPs and thus determine map coverage [49].

For all analyses performed in this manuscript, haplotypes were established following the co-segregation of alleles within families and using computer programmes written by F. Dudbridge and B. Koeleman. Only haplotypes showing definitive parental genotype data are considered in the analyses shown in this paper. In those families with more than one affected patient, only the probands were evaluated.

## Abbreviations

CETDT: conditional ETDT

ETDT: extended transmission disequilibrium test

HM-TDT: haplotype method transmission disequilibrium test

LD: linkage disequilibrium

*MOG*: Myelin Oligodendrocyte Glycoprotein

MS: Multiple sclerosis

## Competing interests

The author(s) declare that they have no competing interests.

## Authors' contributions

**MGM**^**1 **^participated in the study design, discussed results with **FC**^**3 **^and reviewed the manuscript.**RM**^**1 **^checked all data files for genotyping problems, performed statistical analysis on the data and designed all figures and tables. **GC**^**1 **^set up and supervised the different protocols and methods performed.**MCM**^**1**^, **MR**^**1**^, **LS**^**1 **^and **ES**^**1 **^performed amplification and re-sequencing of all exons and introns. **SC**^**1 **^completed all DNA extraction from peripheral blood. **MAS**^**1 **^optimised DNA concentration for all samples and prepared all aliquots and dilutions. **EC**^**1 **^organised family selection and maintained contacts with them for sample collection. **MBW**^**3 **^assisted in manuscript revision and statistical analysis. **MP**^**2**^, **SS**^**2 **^and **GR**^**2 **^provided useful suggestions and collected DNA for part of the patients studied together with the related clinical data. **FC**^**3 **^supervised the study, analysed the results and wrote the manuscript. All authors read and approved the final manuscript.

## Supplementary Material

Additional file 1 TDT results for Alleles of the *MOG *haplotypes treated separately or with *DRB1-DQB1*. Transmission test for linkage disequilibrium was conducted with each of the alleles for the *MOG *haplotypes. The column on the left shows the results for each allele singly, while the column on the right shows the transmission results for *HLA DRB1-DQB1 *when the *MOG *allele is fixed. Click here for file
