# Supplementary material for: Variation of the Myelin Oligodendrocyte Glycoprotein gene is not primarily associated with multiple sclerosis in the Sardinian population
Source: BMC Genet. 2007 May 17;8:25. doi: 10.1186/1471-2156-8-25 (PMC1888712; doi:10.1186/1471-2156-8-25)
Supplement: Additional file 1 — TDT results for Alleles of the MOG haplotypes treated separately or with DRB1-DQB1. Transmission test for linkage disequilibrium was conducted with each of the alleles for the MOG haplotypes. The column on the left shows the results for each allele singly, while the column on the right shows the transmission results for HLA DRB1-DQB1 when the MOG allele is fixed. [file 1471-2156-8-25-S1.doc]

Additional file 1

| Single Point TDT data of the 8 MOG variants associated with multiple sclerosis | | | | |  | TDT data of DRB1-DQB1-MOG haplotypes and pairwise D’ values between DRB1-DQB1 haplotypes and MOG alleles | | | | | | |
| --- | --- | --- | --- | --- | --- | --- | --- | --- | --- | --- | --- | --- |
| SNP07(novel) | T | NT | %T | p |  | DRB1-DQB1 | SNP07 (Novel) | T | NT | %T | p | D’ |
| G | 140 | 89 | 61.1 | 7.5E-04 |  | 0301-0201 | G | 106 | 59 | 64 | 7.2E-04 | 0.7 |
|  |  |  |  |  |  | 0405-0301 | G | 3 | 0 | 100 | 8.3E-02 | -0.8 |
|  |  |  |  |  |  | 1303-0301 | G | 2 | 0 | 100 | 1.6E-01 | -0.4 |
|  |  |  |  |  |  | 0405-0302 | G | 5 | 1 | 83 | 1.0E-01 | -0.4 |
|  |  |  |  |  |  | 1501-0602 | G | 0 | 0 |  |  |  |
|  |  |  |  |  |  | *XXX | G | 16 | 22 | 42.1 | 3.3E-01 |  |
| MOG51 | T | NT | %T | p |  | DRB1-DQB1 | MOG51 | T | NT | %T | p | D’ |
| 226 bp | 199 | 153 | 56.5 | 1.4E-02 |  | 0301-0201 | 226 bp | 96 | 57 | 63 | 2.7E-03 | 0.4 |
|  |  |  |  |  |  | 0405-0301 | 226 bp | 30 | 5 | 86 | 2.4E-05 | 0.4 |
|  |  |  |  |  |  | 1303-0301 | 226 bp | 6 | 2 | 75 | 1.6E-01 | 0.1 |
|  |  |  |  |  |  | 0405-0302 | 226 bp | 10 | 7 | 59 | 4.7E-01 | -0.2 |
|  |  |  |  |  |  | 1501-0602 | 226 bp | 2 | 1 | 67 | 5.6E-01 | -0.6 |
|  |  |  |  |  |  | XXX | 226 bp | 80 | 124 | 39.2 | 2.1E-03 |  |
| SNP06(novel) | T | NT | %T | p |  | DRB1-DQB1 | SNP06 (novel) | T | NT | %T | p | D’ |
| A | 131 | 89 | 59.5 | 4.6E-03 |  | 0301-0201 | A | 100 | 59 | 63 | 2.3E-03 | 0.7 |
|  |  |  |  |  |  | 0405-0301 | A | 3 | 0 | 100 | 8.3E-02 | -0.8 |
|  |  |  |  |  |  | 1303-0301 | A | 2 | 0 | 100 | 1.6E-01 | -0.4 |
|  |  |  |  |  |  | 0405-0302 | A | 5 | 1 | 83 | 1.0E-01 | -0.2 |
|  |  |  |  |  |  | 1501-0602 | A | 0 | 0 |  |  |  |
|  |  |  |  |  |  | XXX | A | 14 | 23 | 29.4 | 1.4E-01 |  |
| rs2071652 | T | NT | %T | p |  | DRB1-DQB1 | rs2071652 | T | NT | %T | p | D’ |
| C | 82 | 47 | 63.6 | 2.1E-03 |  | 0301-0201 | C | 174 | 94 | 65 | 1.7E-06 | 0.6 |
|  |  |  |  |  |  | 0405-0301 | C | 46 | 20 | 70 | 1.4E-03 | 1.0 |
|  |  |  |  |  |  | 1303-0301 | C | 10 | 7 | 59 | 4.7E-01 | 0.0 |
|  |  |  |  |  |  | 0405-0302 | C | 33 | 23 | 59 | 1.8E-01 | 0.5 |
|  |  |  |  |  |  | 1501-0602 | C | 17 | 10 | 63 | 1.8E-01 | 0.7 |
|  |  |  |  |  |  | XXX | C | 106 | 197 | 34.9 | 1.7E-07 |  |
| rs2857767 | T | NT | %T | p |  | DRB1-DQB1 | rs2857767 | T | NT | %T | p | D’ |
| C | 45 | 23 | 66.2 | 7.6E-03 |  | 0301-0201 | C | 171 | 92 | 65 | 1.9E-06 | 0.4 |
|  |  |  |  |  |  | 0405-0301 | C | 48 | 19 | 72 | 4.0E-04 | 0.7 |
|  |  |  |  |  |  | 1303-0301 | C | 1 | 0 | 100 | 3.2E-01 | 0.0 |
|  |  |  |  |  |  | 0405-0302 | C | 33 | 17 | 66 | 2.4E-02 | 0.4 |
|  |  |  |  |  |  | 1501-0602 | C | 17 | 10 | 63 | 1.8E-01 | 1.0 |
|  |  |  |  |  |  | XXX | C | 90 | 209 | 30.1 | 5.9E-12 |  |
| SNP04 (novel) | T | NT | %T | p |  | DRB1-DQB1 | SNP04 (novel) | T | NT | %T | p | D’ |
| G | 108 | 80 | 57.4 | 4.1E-02 |  | 0301-0201 | G | 94 | 57 | 62 | 5.2E-03 | 0.6 |
|  |  |  |  |  |  | 0405-0301 | G | 3 | 1 | 75 | 3.2E-01 | -0.7 |
|  |  |  |  |  |  | 1303-0301 | G | 2 | 0 | 100 | 1.6E-01 | -0.4 |
|  |  |  |  |  |  | 0405-0302 | G | 4 | 3 | 57 | 7.1E-01 | -0.2 |
|  |  |  |  |  |  | 1501-0602 | G | 0 | 0 |  |  |  |
|  |  |  |  |  |  | XXX | G | 18 | 33 | 35.2 | 4.5E-02 |  |
| rs2535260 | T | NT | %T | p |  | DRB1-DQB1 | rs2535260 | T | NT | %T | p | D’ |
| C | 120 | 87 | 58.0 | 2.2E-02 |  | 0301-0201 | C | 163 | 91 | 64 | 1.1E-05 | 0.5 |
|  |  |  |  |  |  | 0405-0301 | C | 44 | 20 | 69 | 2.7E-03 | 0.9 |
|  |  |  |  |  |  | 1303-0301 | C | 9 | 3 | 75 | 8.3E-02 | -0.2 |
|  |  |  |  |  |  | 0405-0302 | C | 29 | 19 | 60 | 1.5E-01 | 0.5 |
|  |  |  |  |  |  | 1501-0602 | C | 12 | 7 | 63 | 2.5E-01 | -0.2 |
|  |  |  |  |  |  | XXX | C | 119 | 202 | 37.1 | 3.6E-06 |  |
| SNP03 (novel) | T | NT | %T | p |  | DRB1-DQB1 | SNP03 (novel) | T | NT | %T | p | D’ |
| A | 135 | 92 | 59.5 | 4.3E-03 |  | 0301-0201 | A | 96 | 54 | 64 | 1.3E-03 | 0.7 |
|  |  |  |  |  |  | 0405-0301 | A | 3 | 0 | 100 | 8.3E-02 | -0.7 |
|  |  |  |  |  |  | 1303-0301 | A | 2 | 0 | 100 | 1.6E-01 | -0.3 |
|  |  |  |  |  |  | 0405-0302 | A | 5 | 0 | 100 | 2.5E-02 | -0.2 |
|  |  |  |  |  |  | 1501-0602 | A | 0 | 0 |  |  |  |
|  |  |  |  |  |  | XXX | A | 11 | 20 | 35.5 | 1.1E-01 |  |

* XXX = all the other *DRB1-DQB1* haplotypes grouped
